# Association of clinicopathological features and prognosis of TERT alterations in phyllodes tumor of breast

**DOI:** 10.1038/s41598-018-22232-w

**Published:** 2018-03-01

**Authors:** Julia Y. S. Tsang, Yau-Kam Hui, Michelle A. Lee, Maribel Lacambra, Yun-Bi Ni, Sai-Yin Cheung, Cherry Wu, Ava Kwong, Gary M. K. Tse

**Affiliations:** 1 Department of Anatomical and Cellular Pathology, Prince of Wales Hospital, The Chinese University of Hong Kong, Ngan Shing Street, Shatin, NT Hong Kong; 2 Department of Pathology, Tuen Mun Hospital, Tuen Mun, Hong Kong; 3 Department of Pathology, North District Hospital, Sheung Shui, Hong Kong; 4 Department of Surgery, Li Ka Shing Faculty of Medicine, The University of Hong Kong, Hong Kong, Hong Kong

## Abstract

Phyllodes tumor (PT) of the breast is a rare but clinically important fibroepithelial tumor with potential risks of recurrence and metastasis. Recent studies identified recurrent TERT promoter mutations in PTs. However, the clinical significance of this alteration has not been fully examined. Two hundred and seven PTs from two intuitions were included. All cases were subjected to immunohistochemical analysis for TERT expression. Analysis of TERT promoter mutations was further performed by Sanger sequencing targeting the hotspot mutation region on cases from one of the involved institutions. The expression of TERT was correlated with clinicopathologic features, mutation status and recurrence. There was an association of TERT expression and its promoter mutation. Both stromal TERT expression and its promoter mutation correlated with PT grading and older patient age. Recurrence free survival (RFS) of PT patients with high stromal TERT expression was shorter if the excision margin was positive. Our findings suggested a possible pathogenic role of TERT alteration in PT malignancy. Currently there is no consensus for re-excision for PT patients with positive surgical margin, particularly for low grade cases. Stromal TERT expression could be potentially useful to guide management patients with benign PTs.

## Introduction

Phyllodes tumor (PT) of the breast is a rare but clinically important fibroepithelial tumor, accounting for 0.3–0.5% of breast tumors^1^. Histologically, PTs are graded as benign, borderline or malignant basing on a combination of histologic criteria^2^. Accurate grading of PT is clinically relevant. While local recurrence of PTs may occur in all grades, metastasis is mostly limited to malignant and few borderline cases^3^. Treatment is mainly surgical as PTs do not response well to systemic therapy^4^.

Recently, studies using massive parallel sequencing identified a number of driver alterations with therapeutic relevance and alterations with diagnostic and prognostic significance in different cancers^5^. For PTs, high frequency of *MED12* mutations affecting exon 2 was reported^6–8^. In addition, recurrent somatic mutations, albeit in lower frequency, have been described for other genes including *RARA, FLNA2, SETD2 and KMT2D*^8^. This mutational landscape was also associated with PT grading. Common somatic mutations affecting cancer associated genes, including *TP53, RB1* and *EGFR* occur exclusively in high grade PTs^6–9^. In addition, frequent mutations in non-coding region of telomerase reverse transcriptase (*TERT*) promoter has also been described^7,10,11^.

Telomerase activation is a known hallmark of cancer^12^. The transcriptional regulation of *TERT* is a decisive factor for controlling telomerase activity. *TERT* promoter mutations are the most common non-coding mutations in cancers^13^. The mutations in the promoter occurred mainly at −124 and −146 bp positions from ATG start site, conferring enhanced promoter activity by putatively generating a consensus binding site (GGAA) for ETS transcription factors^14–16^. *TERT* reactivation/ promoter mutation has been reported to be associated with tumor aggressiveness and poor patient outcome in thyroid^17^ and urothelial^18^ cancers.

*TERT* promoter mutation was described recently in PT^7,10,11^, but its relationship with TERT expression has not been evaluated. Moreover, these studies included small number of cases with limited analysis on their clinicopathologic association. In the present study, the expression of TERT and its promoter mutation in PTs was investigated. Their potential association with each other, clinicopathologic features and patient outcome was evaluated.

## Results

**Figure 1:**
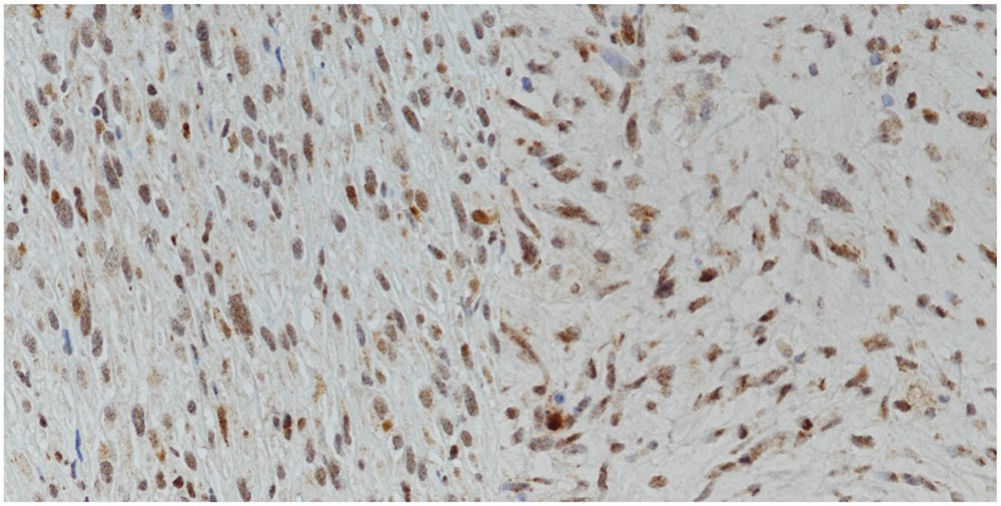
Representative immunohistochemical staining of TERT in stromal components.

Two hundred and seven PTs were included in this cohort. Patients’ age ranged from 16 to 86 years (mean 43 years, median 44 years). Tumor size ranged from 12 to 250 mm (mean 59.6 mm, median 45 mm). Histologically, there were 134 benign (64.7%), 49 borderline (23.7%) and 24 malignant (11.6%) PTs. Post-surgical adjuvant therapy information was available in 178 patients, among them, 30 patients received radiotherapy and three were treated with chemotherapy. Details of the clinico-pathologic features were summarized in Table 1. Overall, TERT staining could be assessed in the stromal component in all 207 cases and in the epithelial component in 197 cases. The mean TERT expression scores in the stromal and epithelial components in the entire cohort were 54.9 ± 41.9 (median = 50; interquartile range (IQR) = 25–70) and 144.3 ± 61.3 (median = 150; IQR = 95–190) respectively. For benign, borderline and malignant PTs, the mean TERT scores in the stromal component were 52.1 ± 41.9 (median = 50; IQR = 18.1–70.6), 56.6 ± 40.0 (median = 52.5; IQR = 33.8–71.3) and 67.2 ± 46.4 (median = 70.0; IQR = 30.0–101.3) respectively. The cases were classified into groups of low, intermediate and high expression of roughly equal frequency for both stromal and epithelial TERT. There were 65 (31.4%), 67 (32.4%) and 75 (36.2%) cases of low, intermediate and high stromal TERT groups; and 58 (34.5%), 65 (33.0%) and 64 (32.5%) cases of low, intermediate and high epithelial TERT staining. Representative staining was shown in Fig. 1.

**Table 1:** Association of stromal TERT expression with clinico-pathological features.

Features N(%)		Low	Intermediate	High	Total	
Age	Mean	39.0	44.5	45.1	43.0	**0.022**
	SD	12.7	11.6	12.6	12.5	
	Median	40	45	46	44	
	Range	16–62	23–81	17–86		
Tumor size	Mean	63.1	57.9	58.0	59.6	0.953
	SD	49.3	36.5	35.5	40.5	
	Median	45.0	48	50	45	
	Range	20–250	12–220	17–180		
Diagnosis	Benign	47(35.1)	40 (29.8)	47 (35.1)	134	**0.044**
	Borderline	11 (22.5)	23 (46.9)	15 (30.6)	49	
	Malignant	7 (29.1)	4 (16.7)	13 (54.2)	24	
	(Borderline/malignant)	(18)	(27)	(28)	(72)	(0.270)
	Total	65	67	75	207	
Border	Pushing	39 (38.6)	29 (28.7)	33 (32.7)	101	*0.086*
	Focal infiltrative	15 (28.8)	21(40.4)	16(30.8)	52	
	Infiltrative	5 (15.2)	12 (36.4)	16 (48.5)	33	
	Total	39	62	65	186	
Mitotic count	<5	46 (33.5)	40 (29.2)	51 (37.2)	137	**0.004**
	5–9	12 (27.3)	23 (52.3)	9 (20.4)	44	
	>9	7 (26.9)	4 (15.3)	15 (57.7)	26	
	Total	65	67	75	207	
Pleomorphism	Mild	38 (39.5)	30 (31.3)	28 (29.1)	96	*0.068*
	Moderate	24 (25.5)	33 (35.1)	37 (39.4)	94	
	Severe	3 (17.6)	4 (23.5)	10 (58.8)	17	
	Total	65	67	75	207	
Cellularity	Mild	38 (36.2)	32 (30.5)	35 (33.3)	105	0.443
	Moderate	17 (25.0)	26 (38.2)	25 (36.8)	68	
	Severe	10 (29.4)	9 (26.5)	15 (44.1)	34	
	Total	65	69	75	207	
Stromal overgrowth	No	44 (36.7)	36 (30.0)	40 (33.3)	120	**0.037**
	Focal	14 (26.4)	23 (43.4)	16 (30.2)	53	
	Present	7 (20.6)	8 (23.5)	19 (55.9)	34	
	Total	65	67	75	207	
Radiotherapy	No	51 (34.5)	49 (33.1)	48 (32.4)	148	0.884
	Yes	9 (30.0)	11 (36.7)	10 (33.3)	30	
	Total	60	60	58	178	
Chemotherapy	No	60 (34.5)	58 (33.3)	56 (32.2)	174	0.367
	Yes	0	2 (66.7)	1 (33.3)	3	
	Total	60	60	57	177

### Clinico-pathologic correlation of stromal TERT expression

Stromal TERT expression was associated with increased patients’ age (p = 0.022), PT grade (p = 0.044), mitotic count (p = 0.004) and stromal overgrowth (p = 0.037). Higher TERT expression also showed a trend of association with infiltrative border (p = 0.086) and increased pleomorphism (p = 0.068). No significant correlation of stromal TERT was found with tumor size, increased cellularity and post-surgical adjuvant therapy (Table 1). Additionally, there were seven cases with necrosis and no association was found with TERT alterations. Due to the limited case number, a conclusive result cannot be reached. Multivariate analysis demonstrated that age (p = 0.009) and stromal overgrowth (p = 0.018) were the independent factors associated with stromal TERT expression (Supplementary Table S1).

### Clinico-pathological correlation of *TERT* promoter mutation

**Figure 2:**
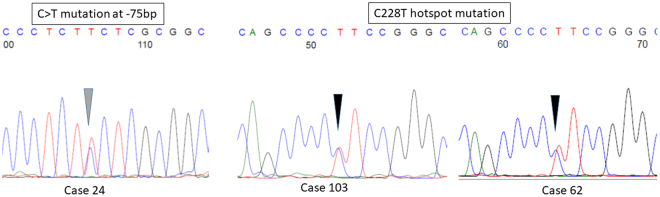
Representative chromatograms of TERT mutations in PTs.

One hundred and four cases from one of the involved institutions were available for *TERT* promoter mutation analysis. Among them, 96 cases had interpretable results from PCR and Sanger sequencing. Overall, 70 cases (72.9%) were wild type and 26 cases (27.1%) showed *TERT* promoter mutation. Except in one case with C > T mutation at −75 bp upstream of start codon, all other cases had hotspot C228T mutation (Fig. 2). The presence of *TERT* promoter mutations was related to older age (p = 0.053); but statistical significance was not reached. The mutation status was not associated with tumor size. Borderline case showed that highest mutation frequency (40%; 10/25 cases), followed by malignant (28.5%; 4/14 cases) and benign (21.1%; 12/57) tumors. For other histologic features, *TERT* promoter mutation was associated with stromal overgrowth (p = 0.032), but not with infiltrative border, increased mitotic count, cellularity or pleomorphism (Table 2). The mutational status correlated with stromal but not epithelial TERT expression. PTs with higher number of mutant correlated showed high stromal TERT expression compared to those with low/intermediate mutant level (p = 0.042). High stromal TERT remained independently associated with promoter mutation in multivariate analysis (Supplementary Table S2). Such association was mainly observed in borderline cases (p = 0.024), but not in benign and malignant cases (Table 2).

**Table 2:** Association of *TERT* promoter mutation with clinico-pathological features and TERT expression.

Features N(%)		WT	Mutant	Total	P-value
Age	Mean	40.7	46.3	42.2	*0.053*
	SD	14.3	9.6	13.3	
	Median	41.0	45.5	43.0	
	Range	16–86	33–69		
Tumor size	Mean	53.1	71.6	58.3	0.134
	SD	36.3	59.8	44.6	
	Median	45.0	52.5	45.0	
	Range	12–220	17–280		
Diagnosis	Benign	45 (78.9)	12 (21.1)	57	0.204
	Borderline	15 (60.0)	10 (40.0)	25	
	Malignant	10 (71.4)	4 (28.6)	14	
	(Borderline/malignant)	25	14	39	(0.108)
	Total	70	26	96	
Border	Pushing	38 (80.9)	9 (19.1)	47	0.161
	Focal infiltrative	15 (60.0)	10 (40.0)	25	
	Infiltrative	15 (71.4)	6 (28.6)	21	
	Total	68	25	93	
Mitotic count	<5	46 (76.7)	14 (23.3)	60	0.547
	5–9	15 (65.2)	8 (34.8)	23	
	>9	9 (69.2)	4 (30.8)	13	
	Total	70	26	96	
Pleomorphism	Mild	43 (72.9)	16 (27.1)	59	0.887
	Moderate	21 (75.0)	7 (25.0)	28	
	Severe	6 (66.7)	3 (33.3)	9	
	Total	70	26	96	
Cellularity	Mild	40 (75.5)	13 (24.5)	53	0.597
	Moderate	17 (65.4)	9 (34.6)	26	
	Severe	13 (76.5)	4 (23.5)	17	
	Total	70	26	96	
Stromal overgrowth	No	51 (81.0)	12 (19.0)	63	**0.032**
	Focal	8 (50.0)	8 (50.0)	16	
	Present	11 (64.7)	6 (35.3)	17	
	Total	70	25	96	
All					
Stromal IHC	Low	30 (76.9)	9 (23.1)	39	0.122
	Intermediate	24 (80.0)	6 (20.0)	30	
	(Low/Intermediate)	54	15	69	(**0.042**)
	High	13 (56.5)	10 (43.5)	23	
	Total	67	25	92	
Epithelial IHC	Low	32 (72.7)	12 (27.3)	44	0.989
	Intermediate	18 (72.0)	7 (28.0)	25	
	(Low/ Intermediate)	50	19	69	(0.892)
	High	17 (73.9)	6 (26.1)	23	
	Total	67	25	92	
Benign					
Stromal IHC	Low	21 (84.0)	4 (16.0)	25	0.636
	Intermediate	13 (76.5)	4 (23.5)	17	
	(Low/Intermediate)	33	8	42	(0.452)
	High	10 (71.4)	4 (28.6)	14	
	Total	44	12	56	
Epithelial IHC	Low	15 (75.0)	5 (25.0)	20	0.862
	Intermediate	15 (78.9)	4 (21.1)	19	
	(Low/Intermediate)	30	9	39	(0.738)
	High	14 (82.4)	3 (17.6)	17	
	Total	44	12	56	
Borderline					
Stromal IHC	Low	5 (55.6)	4 (44.4)	9	**0.024**
	Intermediate	8 (80.0)	2 (20.0)	10	
	(Low/Intermediate)	13	6	19	(**0.012**)
	High	0 (0)	4 (100)	4	
	Total	23	13	36	
Epithelial IHC	Low	10 (71.4)	4 (28.6)	14	0.179
	Intermediate	1 (25.0)	3 (75.0)	4	
	(Low/Intermediate)	11	7	18	(0.618)
	High	2 (40.0)	3 (60.0)	5	
	Total	23	10	23	
Malignant					
Stromal IHC	Low	4 (80.0)	1 (20.0)	5	0.420
	Intermediate	3 (100)	0 (0)	3	
	(Low/Intermediate)	7	1	8	0.510
	High	3 (60.0)	2 (40.0)	5	
	Total	10	3	13	
Epithelial IHC	Low	7 (70.0)	3 (30.0)	10	0.557
	Intermediate	2 (100)	0 (0)	2	
	(Low/Intermediate)	9	3	12	(1.000)
	High	1 (100)	0 (0)	1	
	Total	10	3	13	

### Correlation of TERT expression with recurrence

**Figure 3:**
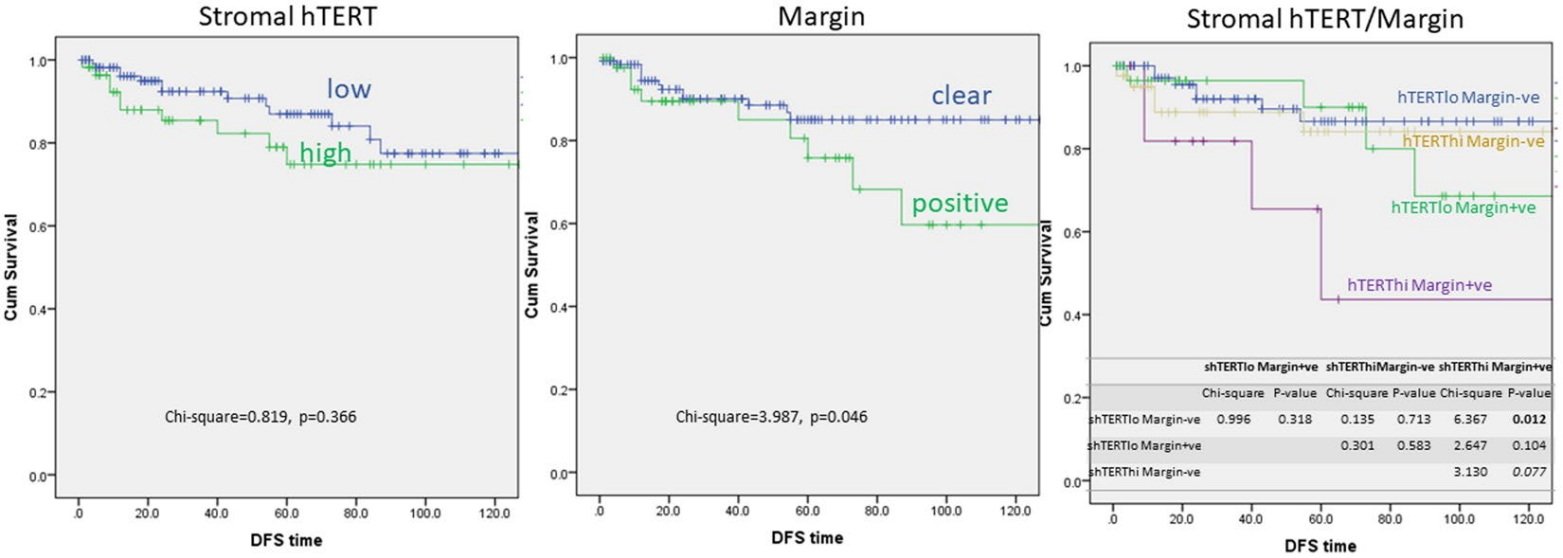
Kaplan-Meier analysis on RFS of PT patients according to (**A**) stromal TERT expression, (**B**) surgical margin status and (**C**) combined analysis of TERT expression and margin status.

Among the 207 cases, 192 cases were primary PTs and outcome data were available for 181 cases. The mean follow-up period was 48 months (median = 38 months, ranged 1–193 months). There were 24 cases with relapse (five cases with distant metastases, 19 cases with local recurrences and one case with both) in which 17 recurrences occurred in the first five years (early relapse). Four cases of death recorded and all of them were preceded by metastatic events. Given the association of high stromal TERT with higher PT grading, its relationship with PT recurrence was examined. Kaplan Meier analysis demonstrated no association between high stromal TERT expression and RFS in PTs (Fig. 3). For the confounding factors, except surgical margin (p = 0.046, Fig. 3), all others factors such as age, size, grade and other histologic factors (border, mitosis, cellularity, pleomorphism, and stromal overgrowth) were not significantly associated with RFS. More intriguingly, those cases with positive margin and high stromal TERT expression demonstrated the worst RFS (Fig. 3) and recurrences from benign PTs. The combined status of high stromal TERT expression and positive margins remained to be an independent prognostic factor for RFS in multivariate analysis (Supplementary Table S3). Of note, cases with high stromal TERT had a trend of early relapse (p = 0.071) in the overall series and were significantly associated with early relapse in cases with positive surgical margin (p = 0.025) (Table 3). However, it did not reach any statistical significance in multivariate analysis for early relapse in cases with clear margin status.

**Table 3:** Association of stromal TERT expression with early relapse (within 5 years).

Groups	Early relapse N(%)	low	Intermediate	High	Total	p-value
All cases	No	54 (33.5)	57 (35.4)	50 (31.1)	161	*0.071*
	Yes	6 (35.3)	2 (11.8)	9 (52.9)	17	
	Total	60	59	59	178	
Clear Margin	No	37 (31.4)	41 (34.7)	40 (33.9)	118	0.247
	Yes	5 (50.0)	1 (10.0)	4 (40.0)	10	
	Total	42	42	44	128	
Involved margin	No	15 (40.5)	14 (37.8)	8 (21.7)	37	**0.025**
	Yes	0 (0)	1 (20.0)	4 (80.0)	5	
	Total	15	15	12	42	

## Discussion

Telomerase reactivation is associated with the acquisition of immortalisation and malignancy in human somatic cells. Its reactivation has been reported in many tumors, including PTs^18^. Telomerase activation has been attributed to several mechanisms. Mutation within the *TERT* promoter region, particularly at two hotspot locations, was suggested to be a common mechanism for telomerase reactivation. In this study, we showed stromal TERT expression in PTs was associated with higher grade, increased stromal mitosis, stromal overgrowth and higher patients’ age. A similar trend was also found with TERT promoter mutation. In addition, TERT promoter mutation could lead to increased TERT expression – a higher frequency of mutation was associated with higher stromal TERT expression, when compared to those with low/intermediate frequency, particularly in borderline PTs. In contrast, there was no correlation between epithelial TERT expression and promoter mutation.

A few studies demonstrated TERT promoter mutation in PT^7,10,11^, however, TERT protein expression in PT has not been studied. We observed *TERT* promoter mutation in 27% of the cases, which was lower than some reported studies (35–65%)^7,11^. It is interesting to note the *TERT* promoter mutation pattern in the current analysis was similar to that reported for Asian population^11^, with a higher prevalence found in borderline cases and the presence of mutations in non-hotspot locations, compared to those from Western populations^7^. Given also the higher prevalence of PT in Asia, the results might possibly reflect the difference in the repertoire of somatic mutations in PTs affecting different ethnicity and differences in PT pathogenesis. The observation of a higher rate of *TERT* mutation in borderline but not malignant PTs may imply the role played by *TERT* mutation occurs early in the malignant progression^19^. *TERT* mutation status showed a significant correlation with stromal TERT expression in borderline cases supporting the potential role of promotor mutation in driving telomerase activation in those cases. In malignant PTs, there was also high stromal TERT expression even though there was a lack of correlation with *TERT* promoter mutation. It is possible that the acquisition of other molecular abnormalities, independent of promoter mutation, in malignant PTs could be sufficient to drive the increased TERT expression. Several factors, including cellular transcriptional factor, hormonal receptors and cytokines, are involved in *TERT* transcription^20^. Some of these factors were shown to be altered in PTs. For instance, c-Myc which was considered to be a major regulator in *TERT* promoter activity^21^, was described to be over-expressed in malignant PTs^22^. On the other hand, p53 which has been shown to repress TERT transcription in a Sp-1 dependent manner^23^, was found to be mutated in malignant PT^8,9^. Recently, a role of histone methylation of TERT regulation has been demonstrated^24^. Of interest, stromal EZH2, an epigenetic modifier, was significantly associated with PT malignancy^25^. Additionally, *TERT* gene amplification was only reported in malignant PTs^7^. Thus, *TERT* upregulation could be part of the molecular mechanism in PT malignant transformation. Chromosomal changes could be found in both epithelial and stromal components of PTs^26^. However, alterations between the two components appear to be distinct. Earlier studies demonstrated that microsatellite instabilities were exclusively found in epithelial components^26^. On the other hand, gene mutations in PT revealed by recent massively parallel sequencing analysis were harboured only in the stromal components^7,8^. In line, *TERT* promoter mutations were reported only in the stromal, not the epithelial components^7,11^. This finding was reflected here with the lack of correlation between its promoter mutation status and epithelial protein expression.

We also observed that stromal TERT expression and its promoter mutation was closely associated with older age. A similar association has been reported in other cancers^27–29^ and PTs^11^. The presence of *TERT* promoter mutations have been detected both in early and late stage of diseases^30^. However, the critical effects of TERT on malignancy may occur at a later time point when the telomeres have become critically short. In fact, recent data showed that *TERT* reactivation may occur at a relatively late stage in molecular carcinogenesis, secondary to the activation of other oncogenic signalling pathways such as MAP kinase signalling in melanoma^31^ or Wnt signalling in hepatocellular carcinoma^15^. With increased age, telomere shortening continued. TERT reactivation could play major roles in preventing crisis when other oncogenic events in PTs took place in aged cells for its malignant progression.

Management of PTs is challenging because of the difficulties in PT diagnosis on core needle biopsy and predicting outcome. Given the correlation of PT grading with its clinical behaviour, it would be helpful in management of patients if PT can be graded in the core needle biopsy. So far, there were no reliable markers that aid diagnosis. On basis of its association with TERT alterations, TERT analysis could be developed as a potential ancillary diagnostic tool, particularly in distinguishing between borderline and benign cases. Surgical margin status is known to impact significantly on the likelihood of PT recurrence^1^. This is confirmed in the current series. About one-fourth of our cases showed positive margin, which was the main significant factor associated with RFS. Interestingly, our results showed that PTs with positive margin and high stromal TERT expression had the worst RFS. It can be explained by the more aggressive nature of tumors with high TERT expression. In routine clinical practice, the management of positive margins after primary excision of PTs varies, with some authors recommending immediate surgery whereas others suggested active monitoring, particularly for low grade lesions^32^. These results suggest the potentials of stromal TERT expression evaluation in the decision making of PT re-excision, and warrant further investigations.

In summary, our data showed an association of TERT expression and its promoter mutation in PT of the breast. Such association mainly occurred in borderline PTs. However, due to the limited cases in subgroup analysis, further examination with a larger cohort will be required. Both stromal TERT expression and its promoter mutation correlated with PT grading and older patient age. Our findings have possible implications in the pathogenic role of TERT alteration in PT malignancy. Despite the relatively low relapse rate of PTs and limited number of cases, we found the RFS of PT patients was worst when the PT showed positive margin AND high stromal TERT expression. As currently there is no consensus for re-excision in PT patients with positive surgical margin, particularly for low grade cases, assessment of stromal TERT expression could be of potential utility to aid surgical management decision making.

## Materials and Methods

PT cases were collected from the archives of the two participating institutions in Hong Kong. Four-micron slides from the formalin fixed paraffin embedded (FFPE) tissue were prepared routinely and stained with hematoxylin and eosin (H&E) stain. All the slides were reviewed first by one of the co-authors (YN or SC) followed by an experienced breast pathologist with expertise in phyllodes tumor (GT) for the following histologic parameters: 1. stromal cellularity; 2. nuclear pleomorphism; 3. stromal overgrowth; 4. mitotic rate; and 5. margin of the tumor, whether infiltrative or rounded. Stromal cellularity and nuclear pleomorphism were graded as 1, 2 and 3 representing low/mild, moderate or high/ severe respectively; stromal overgrowth was graded as absent [presence of epithelial elements within a low power field (x40, Nikon Labophot, field area 1.9 mm^2^)] or present (absence of epithelial element within a low power field). Mitotic count was denoted as the number of mitotic figures per 10 high power fields (hpf) (x400, Nikon Labophot, field area 0.19 mm^2^. The PTs were graded into benign, borderline or malignant using WHO criteria^2^. Patients’ age, tumor size, and outcome data were retrieved from the medical records. Recurrence free survival (RFS) was defined as the duration from the date of initial diagnosis to the first detection of relapse (including both local recurrence and metastatic recurrence). Research was approved by the Joint CUHK-NTEC Research Ethics Committee. All experiments were performed in accordance with relevant guidelines and regulations. All archival samples from pathology tissue bank were retrieved after its use for diagnosis retrospectively. The specimens were obtained in ethical manner and no potential harm will be caused to the patients. For patient confidentiality, all samples were coded by laboratory accession number and were non-identifiable. Therefore, the research could be permissible without consent.

### Tissue microarray (TMA) and immunohistochemistry (IHC)

TMA blocks containing representative epithelial and stromal regions were constructed with quadruple 0.6 mm tissue cores. One section from each TMA was stained with H&E and reviewed to confirm the presence of representative PT.

Immunohistochemical staining of TERT (Alpha Diagnostic International, polyclonal, 1:100, antigen retrieval with Ventana CC1; Incubation room temperature one hour) was performed on the TMA using Optiview Universal DAB Detection Kit (Ventana, Arizona, USA) after deparaffinization, rehydration and antigen retrieval and counterstained with haematoxylin. The validity of the antibody has been reported for western blotting and immunostaining^33^. The sections were scored for the presence and intensity of nuclear staining in the stromal and epithelial cells separately. An immunoscore was obtained by multiplying the staining intensity (graded from 0 to 3, with 0 being no staining, 1 being weak staining, 2 being moderate staining and 3 being strong staining) and the proportion of stained cells (expressed as actual percentage). The immunoscore ranged from 0–300. TERT was classified into three equal groups according to the frequency of expression. Low stromal TERT was scored if immunoscore was ≤30, intermediate expression was scored for immunoscore 31–60 and high level for immunoscore >60. For its epithelial expression, cases with immunoscore ≤120 were classified as low expression while cases with immunoscore 121–169 and ≥170 were classified as intermediate and high expression respectively.

### Extraction of DNA and sequencing of *TERT* promoter

Two 5 micron thick unstained slides were obtained from representative blocks of FFPE samples. Tumor areas were dissected from the section. Adjacent non-tumor tissues were removed using a sterile blade. Genomic DNA was isolated using QIAamp DNA FFPE Tissue Kit (Qiagen) according to manufacturer’s instruction. The concentration of DNA was determined using a NanoDrop ND-1000 Spectrophotometer (NanoDrop Technologies).

PCR was performed in 10-μl reactions containing 100 ng template DNA, 0.3 mM of each dNTP, 2.5 mM MgCl_2_, 0.3 *μ*M of each primer, and 0.2 U of KAPA HiFi HotStart DNA Polymerase (Kapa Biosystems Wilmington, DE, USA), and was initiated at 95 °C for 5 min, followed by 40–45 cycles of 98 °C for 20s, 68 °C for 15s and 72 °C for 30s, and a final extension of 72 °C for 1 min. Primer pairs used to amplify the 163 bp fragment spanning the two mutational hotspots (chr5, 1 295 228 (C228T, −124 bp from ATG) and 1 295 250 (C250T, −146 bp from ATG)) in *TERT* promoter region were as follows: TERT-F (5′-GTCCTGCCCCTTCACCTT-3′) and reverse primer TERT-R (5′-CAGCGCTGCCTGAAACTC-3′). PCR products were subjected to electrophoresis on 1.5% agarose gel and visualised by Molecular Imager Gel Dic XR+ System with Image Lab 4.1 software (Bio-Rad). The PCR products with single band of correct size were sent to BGI-HK for sequencing. The samples were regarded as mutation positive if a mutation was detected in both forward and reverse directions, together with a manual review of chromatograms.

### Statistical Analysis

Statistical analysis was performed using SPSS 23.0 software (SPSS Chicago, IL). Correlation analysis between TERT expression/mutation and clinicopathological parameters were performed using Chi-square test and Fisher’s exact test (when cell size >5). Mann-Whitney U test was used to examine the differences in age and tumor sizes between different stromal TERT expression and promoter mutation status. RFS was estimated with the Kaplan-Meier analysis and two-sides log rank test. Statistical significance was established at p < 0.05.

## Electronic supplementary material

supplementary tables
