# Supplementary material for: Association of clinicopathological features and prognosis of TERT alterations in phyllodes tumor of breast
Source: Sci Rep. 2018 Mar 1;8:3881. doi: 10.1038/s41598-018-22232-w (PMC5832760; doi:10.1038/s41598-018-22232-w)
Supplement: Supplementary file 1 — supplementary tables [file 41598_2018_22232_MOESM1_ESM.pdf]

Association of clinicopathological features and prognosis of TERT alterations in phyllodes tumor of breast

Running title: TERT alterations in breast phyllodes tumor

Julia YS Tsang[1], Yau-Kam Hui[1], Michelle A Lee[1], Maribel Lacambra[1], Yun-Bi Ni[1], Sai-Yin Cheung[2], Cherry Wu[3], Ava Kwong[4], Gary MK Tse[1]*

[1]Department of Anatomical and Cellular Pathology, Prince of Wales Hospital, The Chinese University of Hong Kong, Ngan Shing Street, Shatin, NT, Hong Kong

[2]Department of Pathology, Tuen Mun Hospital, Hong Kong

[3]Department of Pathology, The North District Hospital, Hong Kong

[4]Department of Surgery, Li Ka Shing faculty of Medicine, The University of Hong Kong, Hong Kong

Correspondence to:

Gary M Tse,

Department of Anatomical and Cellular Pathology, Prince of Wales Hospital, Ngan Shing Street, Shatin, Hong Kong.

Tel: (852)35052359

Fax: (852)26374858

Email: garytse@cuhk.edu.hk

Supplementary Table S1 Multivariate linear regression analysis on factors associated with stromal TERT expression

Features	Standard β coefficinent	p-value	Lower 95%CI	Upper 95%CI
Age	0.194	**0.009**	0.003	0.022
Stromal overgrowth	0.175	**0.018**	0.034	0.353

Factors in initial step (backward ward): age, diagnosis, stromal overgrowth, border and mitosis

Supplementary Table S2 Multivariate logistic regression analysis on factors associated with *TERT* promoter mutation

Features	Odd ratio	p-value	Lower 95%CI	Upper 95%CI
High stromal TERT	1.773	**0.024**	1.077	2.918

Factors in initial step (backward ward): age, diagnosis, stromal overgrowth and high stromal TERT expression.

Supplementary Table S3 Multivariate Cox Regression on RFS (with combined analysis on margin and stromal TERT expression)

Features	Hazard ratio	Lower 95% CI	Upper 95% CI	p-value
Age	0.980	0.943	1.017	0.284
Tumor size	1.003	0.992	1.014	0.562
Diagnosis	0.219	0.038	1.262	*0.089*
Stromal overgrowth	1.847	0.927	3.679	*0.081*
Pleomorphism	1.927	0.716	5.184	0.194
Mitosis	2.152	0.534	8.675	0.281
Cellularity	1.365	0.608	3.064	0.451
Border	1.148	0.557	2.369	0.708
hTERTlo Margin-ve	(Reference)			0.169
hTERTlo Margin+ve	2.058	0.515	8.215	0.307
hTERThi Margin-ve	1.463	0.394	5.437	0.570
hTERThi Margin+ve	5.331	1.215	23.395	**0.027**

BOLD: p-value <0.05
